# Improved AlexNet with Inception-V4 for Plant Disease Diagnosis

**DOI:** 10.1155/2022/5862600

**Published:** 2022-09-10

**Authors:** Zhuoxin Li, Cong Li, Linfan Deng, Yanzhou Fan, Xianyin Xiao, Huiying Ma, Juan Qin, Liangliang Zhu

**Affiliations:** School of Integrated Circuit Science and Engineering, Tianjin University of Technology, Tianjin 300384, China

## Abstract

Timely disease detection and pest treatment are key issues in modern agricultural production, especially in large-scale crop agriculture. However, it is very time and effort-consuming to identify plant diseases manually. This paper proposes a deep learning model for agricultural crop disease identification based on AlexNet and Inception-V4. AlexNet and Inception-V4 are combined and modified to achieve an efficient but good performance. Experimental results on the expanded PlantVillage dataset show that the proposed model outperforms the compared methods: AlexNet, VGG11, Zenit, and VGG16, in terms of accuracy and *F*1 scores. The proposed model obtains the highest accuracy for corn, tomato, grape, and apple: 94.5%, 94.8%, 92.3%, and 96.5%, respectively. Also, the highest *F*1 scores for corn, tomato, grape, and apple: 0.938, 0.910, 0.945, and 0.924, respectively, are obtained. The results indicate that the proposed method has promising generalization ability in crop disease identification.

## 1. Introduction

Crop pests and diseases refer to the destruction of normal physiological functions of crops, which can be caused by the invasion of other creatures or environmental changes. As one of the major agricultural disasters, they are characterized by numerous species, high impact, and frequent outbreaks. Crop pests and disease identification are challenging due to the variety of types, the scarcity of technicians in rural areas, and the overreliance on insecticides. Traditional manual pests and disease monitoring rely on observation experience, which inevitably suffers subjectivity and inefficiency. Thus, automatic plant disease detection and control have been the primary concern of each country, especially in recent years, when due to the population increase, food demand is growing at a faster rate [1]. Therefore, it is of great significance to effectively analyze crop pests and diseases and ensure the use of pesticides. Motivated by a great advance in artificial intelligence, crop disease identification also can be conducted using deep learning models [2].

This paper exploits the deep learning model for plant disease recognition, motivated by the great success of deep learning techniques in other applications. The contribution of the research is summarized as follows:

1. A large convolution kernel is used to obtain a large receptive field, enabling the model to focus more on shape than texture. Also, thanks to the employed large kernel, the depth of the model can be compact, which avoids the high computational complexity of the optimization process.
2. Two complementary network structures, Inception-V4 and AlexNet, are combined to take advantage of both networks. The superposition of Inception-X (*X* represents *A*, *B*, *C*) modules in Inception-V4 is removed, which greatly reduces training and inference time with a sacrifice of negligible performance.
3. The dataset is expanded to improve the generalization ability of the model.

The remaining paper consists of the following parts.  Section 2 discusses the current status and limitations of existing research.  Section 3 describes the experimental dataset, and Section 4 describes the proposed network structure, including the loss function and optimizer.  Section 5 presents the experimental results and the analysis, verifying the feasibility of the proposed method. Lastly, this paper is concluded in Section 6.

## 2. Related Works

Multiple plant disease identification has been actively studied, including cassava, guava, and soybeans. Almadhor et al. [3] proposed an AI-based detection framework to classify the common guava fruit diseases. Alli et al. [4] proposed a deep residual convolutional neural network (DRNN) employing different block processing, where the unbalanced dataset was balanced, and gamma correction and decorrelation stretching were used to enhance the color separation of images with significant interband correlations. The DRNN outperformed the plain convolutional neural network (PCNN) on the cassava disease dataset from Kaggle [4]. The modified MobileNetV2 [5] showed a significant improvement in cassava leaf disease recognition on lower-quality images. Ozguven and Adem [6] proposed to detect the disease area of sugar beet leaf via adjusting Region-CNN (R–CNN), which was limited to a small number of sample images.

With the development of smart agriculture, more and more scholars have begun to study the identification of different diseases in different crops. The following papers conducted experiments for crop identification, including apples, corn, and tomatoes.

Srdjan et al. proposed a plant diseases detection model based on the CaffeNet model to identify 13 different types of sick leaves and distinguish leaves from their surroundings [7]. Mohanty et al. evaluated the applicability of AlexNet and GooleNet in a one-leaf multiimage problem [8], where the plant disease diagnosis system on mobile devices was developed and analyzed on the PlantVillage dataset. Geetharamani and Arun Pandian proposed a nine-layer DCNN for disease identification [9]. In [10], Triki et al. proposed a leaf detection and segmentation model, deep leaf, which was based on Mask-RCNN and used morphological characteristics in plant specimens. Liu et al. applied a long short-term memory network-based variational autoencoder to extract the sequential feature of the application running time [11]. Rao et al. used bilinear convolutional neural networks (bi-CNNs) for identifying different types of leaves, where VGG and ResNet were used as feature extractors [12]. Dyrmann et al. constructed a convolutional neural network (CNN) to distinguish seedlings in different stages of growth. However, due to the small number of data samples, the network suffered from low classification accuracy [13]. Ferreira et al. proposed a detection model and constructed a dataset for weed detection in soybean crops [14]. Ghazi et al. applied a pretrained AlexNet, GoogletNet, and VGGNet to classify plant species in a dataset of given unconstrained photos, showing that the primary factor that affects the performance of fine-tuning was the iterations number [15]. Liu et al. proposed an end-to-end pest detection network, PestNet, where the Channel–Spatial Attention module was used to extract high-quality features for large-scale diseases [16]. Chao et al. proposed XDNet based on deep separable convolution and dense connection structure to identify apple leaf disease [17]. The XDNet used normalization and data enhancement to avoid overfitting and improve the stability of the network. Valeria et al. assessed the classification accuracy of tomato plant diseases based on AlexNet, GoogleNet, Inception V3, ResNet18, and ResNet 50 [18]. The results showed that GoogleNet is superior to other architectures in terms of accuracy, and AlexNet is the fastest model. Guo et al. proposed a plant disease detection and recognition model based on the RPN algorithm containing the feature of symptoms through the Chan–Vese (CV) algorithm [19].

Many studies have been conducted to improve recognition accuracy by optimizing the structure of deep learning networks, including batch normalization, dropout, and replacement of fully connected layers with pooling layers.  Table 1 shows the summary of related studies with highlights of the proposed methods for data augmentation and classification. Some shortfalls of the existing plant disease classification models include data scarcity and class. Also, many works were focused on the efficient structure while avoiding overfitting, including dataset augmentation with mirroring, rotation, and additive noise. Among many deep learning models, AlexNet is one of the widely used models, mainly due to its simplicity, to identify defects of various crops and judge the speed of the germination process. The AlexNet network model is also widely used in agriculture, medicine, and power engineering fields.

Three types of convolution kernels are used in the Inception-V4 model: 7^*∗*^7, 5^*∗*^5, and 3^*∗*^3. In this paper, a larger convolution kernel is used instead of increasing the depth of the network for more features. Also, in order to further reduce the depth of the network, this paper removes the superposition of Inception-X (*X* represents *A*, *B*, and *C*) modules in Inception-V4. Accordingly, the depth of the network is greatly reduced, shortening the training time as a consequence.

For the two reasons above, this paper combines two networks: AlexNet and Inception-V4, to identify diseases in corn, tomato, grape, and apple crops. Specifically, healthy, CercosporaGrayspot, Commonrust and NorthernBlight in corn, healthy, Bacterialspot, Lateblight and Septorialspot in tomato, Blackrot, healthy, Blackrot, sariopsisSpot and Measles in grape, healthy, Blackrot, Cedarrust and scab in apple. Experiments on the PlantVillage dataset show that the proposed model outperforms four compared networks in terms of identification accuracy. Further, the dataset is expanded to improve the generalization ability of the model to avoid overfitting caused by the small number of training samples, which is especially of significance in plant disease identification.

## 3. Dataset Processing

### 3.1. Dataset

**Figure 1:**
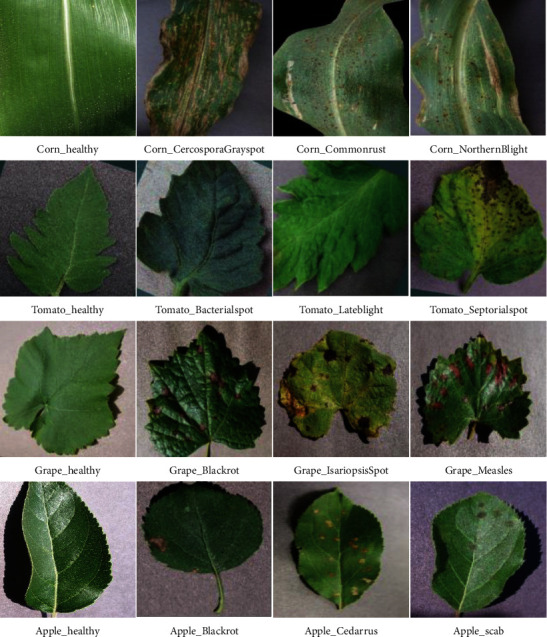
Plant village dataset classification examples.

The PlantVillage Project (https://www.plantvillage.org) is an open-source website for users that addresses all plant diseases [20]. This dataset includes 61,486 images with 39 different categories of plant leaves. In the study, four plant leaves from the PlantVillage dataset are selected as experimental subjects. In order to make the experimental data more universal, the images of plant diseases such as leaf curling, mutilation, and wilting were not discarded. Sixteen diseased and healthy samples of four plants are represented, as shown in Figure 1.

In the dataset, there are 16 types of diseased and healthy plants. The images are divided into training and test sets in an 8 : 2 ratios, whose detailed descriptions are given in Table 2.

### 3.2. Dataset Augmentation

**Figure 2:**
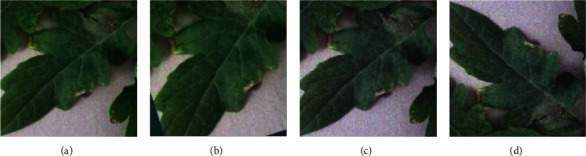
An example of the dataset augmentation. (a) Original image. (b) Affine transformation. (c) Superimposed Gaussian noise. (d) Flip vertically.

The original data from PlantVillage consists of a total of 21,035 images, which is an insufficient amount to train plant leaf diseases without overfitting when considering the number of diseases. For a richer and more generalized dataset, the original dataset was augmented via the affine transformation, superimposed Gaussian noise, and vertical flip [17], as shown in Figure 2.

In this study, each image was rotated 30 degrees counterclockwise and 1.2 times enlarged with an affine transformation. Also, the Gaussian noise (mean = 0, variance = 0.025) was added to the input images, which ensured that the brightness of the image remained unchanged during the shooting process, and the Gaussian noise of the image effectively simulated the noise interference phenomenon in the actual shooting process. Gaussian noise superimposed can reduce the dependence on certain properties and improve the robustness of the model [21]. Furthermore, a vertical flipping was randomly applied for each image. With these data augmentations, the final 168,280 images were obtained.  Table 3 summarizes the quantity of the dataset after each data augmentation process.

### 3.3. Preprocessing

Preprocessing plays a great role in correct classification. First, all images in the dataset are resized to 227^*∗*^227, normalized into the range of [0, 1], and finally standardized based on the arithmetical average (avg) and the standard deviation (std), as follows:

(1) $$\begin{array}{c} \text{Standardized}\_\text{Image}=\frac{\text{image}-\mathrm{avg}}{\mathrm{std}}. \end{array}$$

The preprocessed image has pixel values ranging from −1 to 1. Normalization and standardization reduce the adverse effects caused by singular sample data.

## 4. Network Structure

### 4.1. CNN

CNN has been the most popular deep learning network structure in the image recognition field. The classical networks include AlexNet, VGGNet, and GoogleNet. The advent of AlexNet opened the door to deep learning research, which was the basis of many subsequent deep learning models. The innovation of AlexNet was the employment of the nonlinear unsaturated function, ReLU, instead of the original Softmax function. Local response normalization (LRN) was also used to improve accuracy and generalization capability. Furthermore, a smaller stride was used than the polling size, which improved feature richness and minimized information loss since the outputs of the pooling layers overlapped and covered each other [22]. The first layer of AlexNet uses a large convolution kernel to better extract global information such as location. The front layer is large enough to get a larger receptive field and provide more information for the later layers. Due to a large amount of information, feature mapping and the pixel-by-pixel classifier can be closely connected, thereby enhancing the ability to deal with different transformations [23]. Although the AlexNet has large convolution kernels and deepens the depth of the network, it raises the risk of gradient disappearance and has low accuracy.

GoogleNet introduced the concept of the Inception structure. The GoogleNet proposed a local network structure (Inception module) with strong expressiveness but small computation, which can be stacked. Then, the BN layer and the decomposed network structure were added. This structure improved a significant accuracy with a little computational complexity increase. The Inception-V3 model is the third generation model in the Google Inception series, which utilizes parallel pooling and asymmetric convolution. The stem module of Inception-V4 followed the basic principles in Inception-V3. Compared with the accumulation of simple multi-layer convolution kernel pooling in Inception-V3, Inception-V4 had a simpler architecture and more Inception modules with high accuracy [24].

### 4.2. Improved AlexNet Network Structure

In this paper, the AlexNet structure is combined with the inception-V4 structure to take both cons of those two structures. The Inception-V4 module is added based on the AlexNet structure, strengthening the preprocessing ability of the network. The BN is also applied to accelerate the convergence, improve the generalization ability, and prevent the gradient from disappearing. During the phase of model training, each sample in the batch is normalized by calculating the mean  *μ* and variance *σ*^2^ per batch.

(2) $$\begin{array}{c} \mu =\frac{1}{N}\sum_{i=1}^{N}x_{i},\\ \sigma^{2}=\frac{1}{N}\sum_{i=1}^{N}{\left(x_{i}-\mu \right)}^{2},\\ {\tilde{x}}_{i}=\frac{x_{i}-\mu }{\sqrt{\sigma^{2}+\varepsilon }}, \end{array}$$

where  *N* is batch size and  *x*_*i*_ is a sample in the batch. For a two-dimensional image input, the convolution output of the BN layer is (*N*, *C*, *W*, *H*), where *C* indicates the number of output channels, and *W* and *H* indicate the dimension of the feature map. Then, each sample in the batch can be expressed as *x*_*c*,*w*,*h*_. The BN normalizes each sample separately, so the calculated number of  *μ*  is also *C* × *W* × *H*.

During the inference phase, the BN layer uses the mean $\tilde{\mu }$ and variance ${\tilde{\sigma }}^{2}$ calculated in the training phase, which is computed by the moving average method.

(3) $$\begin{array}{c} \tilde{\mu }=\mu_{n}=\alpha \mu_{n-1}+\left(1-\alpha \right)\cdot \frac{1}{N}\sum_{i=1}^{N}x_{i,n}, \end{array}$$

where *x*_*i*,*n*_ is a sample in the nth batch,  *α* is the step size factor of the learning rate, and *μ*_*n*_ is the mean value obtained when training to the nth batch. Similarly, ${\tilde{\sigma }}^{2}$ is approximated in the same way [25].

Finally, a scaling coefficient *γ* and a translation coefficient *β* are often added to the calculation of BN, and then the output is:

(4) $$\begin{array}{c} y_{i}=\gamma \frac{x_{i}-\mu }{\sqrt{\sigma^{2}+\varepsilon }}+\beta . \end{array}$$

**Figure 3:**
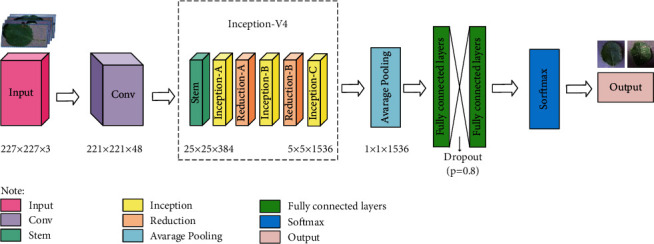
The structure of the improved AlexNet.

The BN layer can speed up the training and convergence of the network, control the gradient explosion, prevent the gradient from disappearing, and avoid overfitting. The structure of the improved AlexNet is depicted in Figure 3.

As shown in Figure 3, the convolution layer first extracts the texture information of the input image from the shallow edge structure to the deep texture semantic structure. Then, the Inception-V4 further extracts features as a backbone network, which consists of multiple convolutions and pooling operations. Inception-X (*X* represents *A*, *B*, and *C*) module learns image features through multiple parallel feature transfer structures, improving the feature utilization. Reduction-X (*X* represents *A*, *B*) module, as a pooling layer, convert large feature maps into small feature maps, where the number of channels increases. In this way, too high computational complexity can be avoided without no significant loss of information [26]. Then, the Average Pooling layer reduces the deviation of the estimated mean, improving the robustness of the model and reducing the number of parameters. Also, the Dropout layer is used in the two fully connected layers to prevent the overfitting problem, where a certain amount of neurons are temporarily discarded from the network during the training process. Lastly, the Softmax regression, as the output layer, maps the results to the (0, 1) probability interval [27]. The use of multiple parallel convolution paths reduces the number of network parameters. Compared with the network without deepening layers, the network with deepening layers can achieve a similar (or better) performance with fewer parameters. The initial layer only needs to focus on learning edge information and can learn efficiently with less training data. By deepening the network, feature information can be decomposed hierarchically, thus improving learning efficiency. The detailed structure of the network is given in Table 4.

Combining different information obtained from different convolution layers obtains a discriminative image representation. Thus, the proposed model adopts convolution kernels of different sizes to extract features, stack the obtained features along the channel dimension, and transmit them to the next layer. The original first layer convolution of AlexNet provides a large amount of data for Inception-V4. It reduces the loss of information to some extent. At the same time, the small convolution kernel and deeper layer in the Inception-V4 network reduce the number of parameters and computation, improving the efficiency, and quality of classification. Dropout and the CrossEntropyLoss function avoid gradient disappearance and overfitting. Finally, the Adam optimizer adjusts the internal parameters of the network by minimizing the loss function.

### 4.3. Dropout and Loss Function

**Figure 4:**
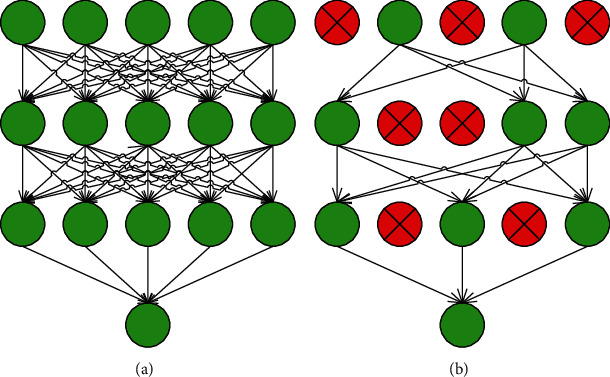
The comparison before and after adding dropout. (a) Without dropout. (b) With dropout.

Dropout aims to improve the generalization capability of the model by inactivating neurons with a particular probability throughout the training process. The use of the Dropout can reduce the dependence on the part of upper neurons. It also prevents overfitting by integrating multiple models with different network structures, as is shown in Figure 4.

The CrossEntropyLoss function is used as a loss function. The CrossEntropy function is often combined with the Softmax function to prevent the gradient from disappearing. It solves the problem of slow or stagnant updates of weights in the hidden layer.

The CrossEntropyLoss function is formulated as:

(5) $$\begin{array}{c} H\left(p,q\right)=-\sum_{i=1}^{M}p\left(x_{i}\right)\log\left(q\left(x_{i}\right)\right), \end{array}$$

where *M* represents the number of categories *p*(*x*_*i*_) and *q*(*x*_*i*_) represent the sample distribution and the prediction distribution, respectively *H*(*p*, *q*). is used to compute the degree of deviation of the ground truth from the output values in the test set. The smaller the result value obtained by the function is, the closer the distribution of *p* and *q* is, and the better the performance is. However, in the backpropagation process, the greater the gap between the ground-truth value and the output value accelerates the parameter adjustment of the model.

### 4.4. Optimizer

**Figure 5:**
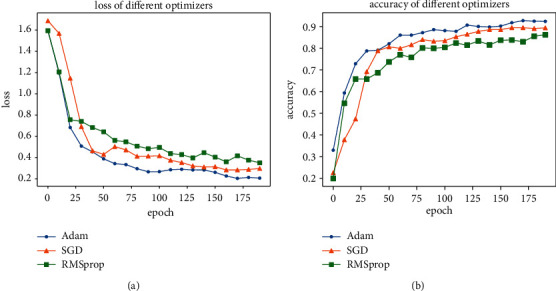
Accuracy and loss comparison of the different optimizers. (a) Loss of different optimizers. (b) Accuracy of different optimizers.

The optimizer can promote the desired loss function by reducing the gradient and calculating the derivative of the multivariate function. Typical optimization algorithm includes adaptive moment estimation (Adam), stochastic gradient descent (SGD), and RMSProp. These three different optimizers were analyzed with the proposed model. The loss and accuracy for each epoch are depicted in Figure 5. Combined with AlexNet and inception-V4, the Adam optimizer adjusts the learning rate of each parameter to prevent the learning rate decay, stabilize the exponential gradient decay, improve the identification accuracy of the network, and reduce the loss.

## 5. Experimental Results and Analysis

### 5.1. Experimental Environment

Experiments were conducted on the computer with the CPU intel core i7 8565U and Windows 11. The models were implemented in Python 3.7.10 and Pytorch deep learning library. The model was trained for 200 epochs with the Adam optimizer and the initial learning rate = 0.0001.

### 5.2. Comparison of Different Network Models

**Figure 6:**
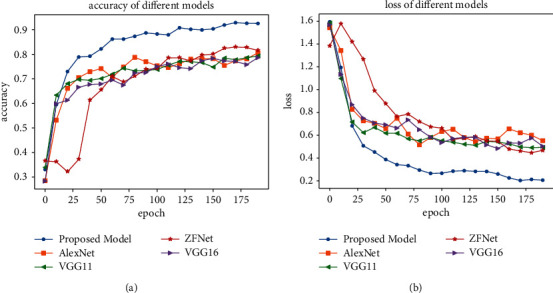
Accuracy and Loss comparison of different network models. (a) Accuracy comparison. (b) Loss comparison.

The proposed model was evaluated with compared methods: AlexNet, VGG11, ZFNet, and VGG16. The loss is used to update the model parameters, while the accuracy is used to evaluate the performance of the model.  Figure 6 compares the accuracy and loss for each epoch of the proposed model and the compared models, showing the superiority of the proposed model over the other models. With the increase in epochs, the proposed model converges better and faster.

### 5.3. Analysis of Dataset Augmentation

**Figure 7:**
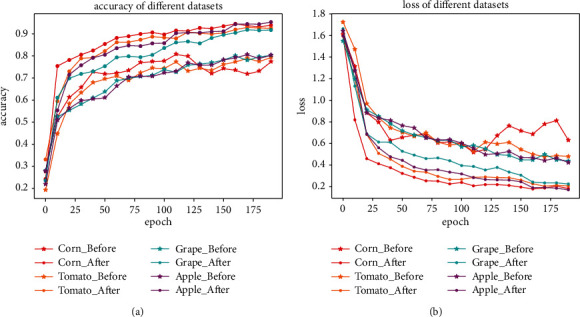
The comparison of accuracy and loss before and after dataset expansion. (a) Accuracy comparison (b) Loss comparison.

In order to alleviate the overfitting problem during the training [17], the dataset was augmented with affine transformation, Gaussian noise, and vertical flip.  Figure 7 compares the accuracy and loss with and without the data augmentation for corn, tomato, grape, and apple data. As shown in Figure 7, the data augmentation significantly improves the performance for all types of data (corn, tomato, grape, and apple): 0.945, 0.948, 0.923, and 0.965 with the data augmentation vs. 0.787, 0.826, 0.854 and 0.828 without the data augmentation.

### 5.4. Comparison of Training Data and Test Data

**Figure 8:**
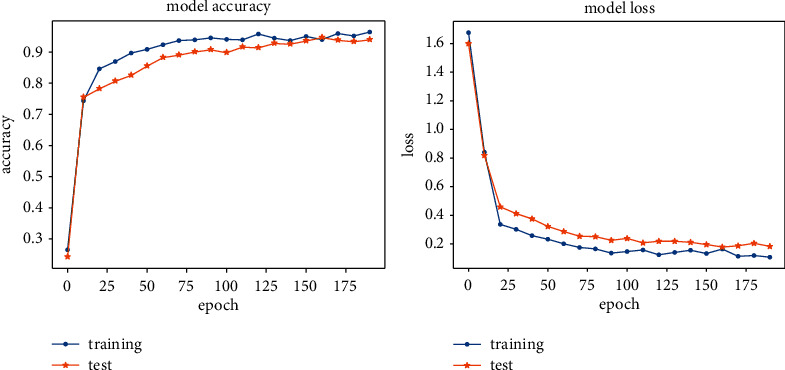
The comparison of the accuracy and loss of the training set and the test set.

Figure 8 compares the accuracy and loss obtained from the training and test datasets, showing that for epochs >60, the accuracy and loss curve have converged. The accuracy of the test data reaches 0.94, and the loss reaches 0.16. The fluctuation degree of the two curves is small. Also, it can be seen that there is little difference between the training set and the test set, proving good adaptability and stability of the proposed model.

### 5.5. Confusion Matrix

**Figure 9:**
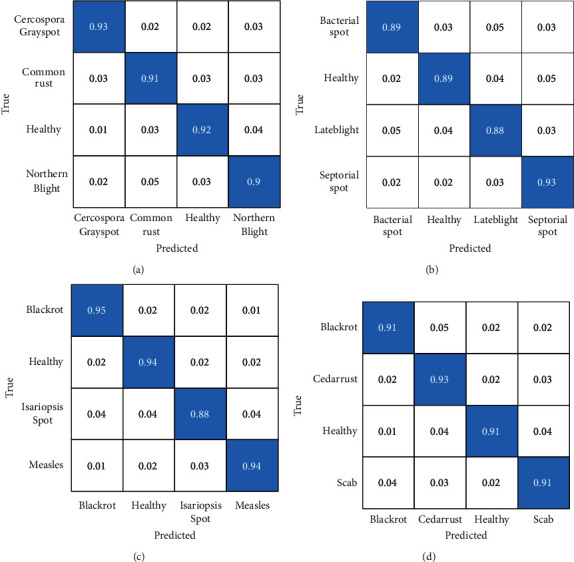
The confusion matrix of four plants (a) Confusion matrix of corn dataset (b) Confusion matrix of tomato dataset (c) Confusion matrix of grape dataset (d) Confusion matrix of apple dataset.

The confusion matrix is mainly used to determine the merits of classifiers. Due to the complexification of the patterns displayed in each class, the system tends to obfuscate in multiclass classification [28].  Figure 9 presents the confusion matrix of the ultimate classification results. The diagonal elements represent the quantity of judged correct and are proportional to the global precision of the training model.

As shown in Figure 9, the recognition accuracy of the grape is the highest (0.95). The Grape_Measles and Grape_Blackrot leaves of grapes are the least easily confused, with a probability of the two being misidentified by only 0.1. The recognition capability of the proposed model can be intuitively assessed through the confusion matrix, which helps further to analyze the confusion degree of various plant disease identification.

The performance of the proposed method is evaluated in terms of accuracy, precision, recall, and *F*1 Score, which are computed based on the true positive (TP), true negative (TN), false positive (FP), and false negative (FN). The calculation formulas are as follows:

(6) $$\begin{array}{c} \text{Accuracy}=\frac{\mathrm{TP}+\mathrm{TN}}{\mathrm{TP}+\mathrm{TN}+\mathrm{FP}+\mathrm{FN}},\\ \mathrm{Precision}=\frac{\mathrm{TP}}{\mathrm{TP}+\mathrm{FP}},\\ \mathrm{Recall}=\frac{\mathrm{TP}}{\mathrm{TP}+\mathrm{FN}},\\ \mathrm{F1Score}=2\frac{\mathrm{Precision\times Recall}}{\mathrm{Precision}+\mathrm{Recall}}. \end{array}$$

In the case of 1,200 samples, accuracy, precision, recall, and *F*1 Score of Corn_healthy are 0.964, 0.927, 0.930, and 0.928, respectively. To further demonstrate the generalization of the proposed model in plant disease identification, relevant parameters in different plant diseases are listed as follows.

Table 5 shows that the top three accuracies were Grape_Measles (0.972), Grape_Blackrot (0.970), and Corn_CercosporaGrayspot (0.970) in a case of 1,200 samples. Meanwhile, its precision and recall reached the level of 0.87 or more. After 200 epochs, the highest accuracy of corn, tomato, grape, and apple reached 0.945, 0.948, 0.923, and 0.965, respectively, and the highest *F*1 scores of corn, tomato, grape, and apple reached 0.938, 0.910, 0.945, and 0.924, respectively.

The analysis and comparison demonstrate the practicability of the proposed model in the plant disease identification and classification field. Extensive experiments show that the proposed model trained on a large-scale plant leaf dataset can obtain accurate and stable results. It indicates that the proposed model can be used to diagnose plant diseases to take action in time and achieve healthy growth of crops.

### 5.6. ROC & AUC

**Figure 10:**
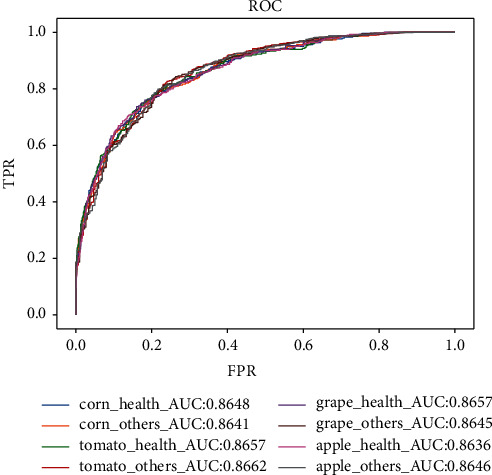
ROC curve.

The receiver operating characteristic (ROC) curve is an analysis tool that is depicted on a two-dimensional plane, where the abscissa of the ROC is false positive rate (FPR), and the ordinate is true positive rate (TPR). The AUC indicates the area under the ROC curve. The TPR and FPR are defined as follows:

(7) $$\begin{array}{c} \mathrm{TPR}=\frac{\mathrm{TP}}{P},\\ \mathrm{FPR}=\frac{\mathrm{FP}}{F}, \end{array}$$

where *P* represents the number of positive samples and *F* represents the number of negative samples. For a classifier, FPR and TPR can be obtained according to their performance on the test sample. The ROC curve and corresponding AUC of the proposed model are shown in Figure 10

## 6. Conclusion

This paper proposes an improved AlexNet with Inception-V4 for plant disease diagnosis, where the AlexNet convolutional layers were appropriately adjusted, and Inception-V4 was added as a backbone network. Extensive experiments on the PlantVillage dataset showed superior performance (accuracy = 0.965) of the proposed method over the compared models: AlexNet, VGG11, VGG16, and ZFNet. Furthermore, analysis of different optimizers and data augmentation were conducted to confirm that the Adam optimizer and data augmentation improved the performance and robustness of the model. Also, the proposed model was evaluated in terms of accuracy, precision, recall, *F*1 Score, ROC, and AUC. The experimental results show that the proposed model performs well in plant disease detection. However, still, the proposed model cannot distinguish different plant diseases in crops that have similar features. No clear boundary between different levels of the same plant disease causes misidentification, leading to the lower identification accuracy of the plant disease. Also, the used dataset is single-leaf oriented upward images with a homogenous background. In the real world, the background is more complex, and some plant diseases do not even appear on the surface of the leaves but exist in other roots, stems, and other parts of the plant. Thus, future works will investigate to address these limitations.

## Figures and Tables

**Table 1:** Summary of related works on plant disease classification.

Reference	Plant types	Dataset	Data augmentation	Methods	Limitation
Srdjan et al. [7]	13 kinds of plants	Stanford background dataset	Image transformations used for augmentation: (a)affine transformations; (b)perspective transformations; (c) rotations.	CNN	Training less data

Mohanty et al. [8]	14 crop diseases	PlantVillage	Resize the images to 256 × 256 pixels, and perform both the model optimization and predictions on these downscaled images	AlexNet	When tested on a set of images taken under conditions different from the train images, the accuracy is reduced substantially to just above 31%

Dyrmann et al. [13]	22 crop samples	BBCH12e16	—	DCNN	Due to the small number of training samples, the recognition accuracy fluctuates greatly

Ferreira et al. [14]	Soybean crops diseases	Captured by the UAV	—	ConvNets or CNNs	Dependency on feature extractors

Ghazi et al. [15]	1,000 species of trees, herbs, and ferns	LifeCLEF 2015	Decrease the chance of overfitting, image transforms such as rotation, translation, reflection, and scaling	GoogleNet, AlexNet, and VGGNet	As an example, increasing the batch size from 20 to 60 increases the training time 3-fold but does not match the performance obtained by increasing the number of iterations by the same amount

Liu et al. [16]	16 kinds of insect pests	Multi-class pest dataset 2018 (MPD2018)	—	CNN	The model did not do a good job of identifying similar pests in different categories methods

Geetharamani and Arun Pandian [9]	13 different of plant leaves	PlantVillage	Image flipping, gamma correction, noise injection, PCA color augmentation, rotation, and scaling transformations	Deep CNN	The model can only identify leaf diseases, but it cannot identify other parts of the plant diseases

Ozguven and Adem [6]	Sugar beet leaf disease	Sugar beet leaf images dataset	—	Faster R–CNN	The accuracy of disease detection is low

Chao et al. [17]	Apple tree leaf diseases	Laboratory independent planting and cultivation	Image scaling, dataset expansion, and dataset normalization	DCNN	There are few types of data sets, and the specific network architecture of various structures lacks a description

**Table 2:** Dataset of plant leaves.

Disease type	Label	Total number of pictures (sheets)	Training set (sheets)	Validation set (sheets)
Corn	Corn_healthy	1162	930	232
	Corn_CercosporaGrayspot	1000	800	200
	Corn_Commonrust	1192	954	238
	Corn_NorthernBlight	1000	800	200

Tomato	Tomato_healthy	1591	1273	318
	Tomato_Bacterialspot	2127	1702	425
	Tomato_Lateblight	1908	1526	382
	Tomato_Septorialspot	1771	1416	355

Grape	Grape_healthy	1000	800	200
	Grape_Blackrot	1180	944	236
	Grape_IsariopsisSpot	1076	860	216
	Grape_Measles	1383	1106	277

Apple	Apple_healthy	1645	1316	329
	Apple_Blackrot	1000	800	200
	Apple_Cedarrust	1000	800	200
	Apple_scab	1000	800	200

**Table 3:** Dataset augmentation process.

	Original data (sheets)	Operations	Final data (sheets)
The first time	21,035	Affine transformation	42,070
The second time	42,070	Superimposed Gaussian noise	84,140
The third time	84,140	Flip vertically	168,280

**Table 4:** The detailed structure of the AlexNet-Inception-V4 network.

Layer name	Tensor size
Input	[3, 227, 227]
Conv	[48, 221, 221]
Stem	[384, 25, 25]
Inception-A	[384, 25, 25]
Reduction-A	[1024, 12, 12]
Inception-B	[1024, 12, 12]
Reduction-B	[1536, 5, 5]
Inception-C	[1536, 5, 5]
Average pooling	[1536, 1, 1]
Fully connected layers	[1536]
Output	[4]

**Table 5:** The accuracy, precision, recall, and *F*1 score for the proposed model.

	Accuracy	Precision	Recall	*F*1 score
Corn_CercosporaGrayspot	**0.970**	0.940	0.937	0.938
Corn_Commonrust	0.955	0.907	0.913	0.910
Corn_healthy	0.964	0.927	0.930	0.928
Corn_NorthernBlight	0.960	0.927	0.900	0.917
Tomato_Bacterialspot	0.948	0.905	0.887	0.896
Tomato_healthy	0.949	0.907	0.887	0.897
Tomato_Lateblight	0.936	0.870	0.873	0.872
Tomato_Septorialspot	0.954	0.891	0.930	0.910
Grape_Blackrot	**0.970**	0.937	0.943	0.940
Grape_healthy	0.964	0.919	0.940	0.929
Grape_IsariopsisSpot	0.953	0.923	0.883	0.903
Grape_Measles	**0.972**	0.944	0.947	0.945
Apple_Blackrot	0.960	0.929	0.910	0.919
Apple_Cedarrust	0.950	0.880	0.927	0.903
Apple_healthy	0.963	0.941	0.907	0.924
Apple_scab	0.956	0.910	0.913	0.912

## Data Availability

The data that support the findings of this study are available from the corresponding author upon reasonable request.
